# HIV pre-exposure prophylaxis was associated with no impact on sexually transmitted infection prevalence in a high-prevalence population of predominantly men who have sex with men, Germany, 2018 to 2019

**DOI:** 10.2807/1560-7917.ES.2022.27.14.2100591

**Published:** 2022-04-07

**Authors:** Hendrik Streeck, Klaus Jansen, Trevor A Crowell, Allahna Esber, Heiko K Jessen, Christiane Cordes, Stefan Scholten, Stephan Schneeweiss, Norbert Brockmeyer, Christoph D Spinner, Markus Bickel, Stefan Esser, Jukka Hartikainen, Albrecht Stoehr, Clara Lehmann, Ulrich Marcus, Jörg Janne Vehreschild, Alexandra Knorr, Anna-Lena Brillen, Carsten Tiemann, Merlin L Robb, Nelson L Michael

**Affiliations:** 1 Institute of Virology, University Hospital, University of Bonn, Bonn, Germany; 2 Institute of HIV Research, University Duisburg-Essen, Essen, Germany; 3 Robert Koch Institute, Berlin, Germany; 4 U.S. Military HIV Research Program, Silver Spring, United States; 5 Henry M. Jackson Foundation for the Advancement of Military Medicine, Bethesda, United States; 6 Private Practice Jessen+Jessen, Berlin, Germany; 7 Private Practice Cordes, Berlin, Germany; 8 Private Practice Hohenstaufenring, Cologne, Germany; 9 WIR-Walk In Ruhr, Center for Sexual Health and Medicine, Bochum, Germany; 10 Interdisciplinary Immunological Outpatient Clinic, Center for Sexual Health and Medicine, Department of Dermatology, Venereology and Allergology, Ruhr University Bochum, Bochum, Germany; 11 Technical University of Munich, School of Medicine, University Hospital Rechts der Isar, Department of Internal Medicine II, Munich, Germany; 12 Infektiologikum Frankfurt, Frankfurt, Germany; 13 HPSTD clinic, University Hospital Essen, University Duisburg-Essen, Essen, Germany,; 14 Private Practice ZIBP, Berlin, Germany; 15 Institute for interdisciplinary medicine, Hamburg, Germany; 16 University of Cologne, Cologne, Germany; 17 Laboratory Krone, Bad Salzuflen, Germany; 18 Walter Reed Army Institute of Research, Silver Spring, United States

**Keywords:** Pre-Exposure Prophylaxis, Sexually Transmitted Diseases, Mycoplasma genitalium, Gonorrhea, Chlamydia, Syphilis

## Abstract

**Introduction:**

Despite increased use of pre-exposure prophylaxis (PrEP) in Germany, HIV infection rates are not declining and little is known about how this prevention method affects the prevalence of sexually transmitted infections (STI) among men who have sex with men (MSM).

**Aim:**

We studied, in a large multicentre cohort, STI point prevalence, co-infection rates, anatomical location and influence of PrEP.

**Methods:**

The BRAHMS study was a prospective cohort study conducted at 10 sites in seven major German cities that enrolled MSM reporting increased sexual risk behaviour. At screening visits, MSM were tested for *Mycoplasma genitalium* (MG), *Neisseria gonorrhoeae* (NG), *Chlamydia trachomatis* (CT) and Treponema pallidum *(TP)*, and given a behavioural questionnaire. With binomial regression, we estimated prevalence ratios (PR) and 95% confidence intervals (CI) for the association of PrEP and STI.

**Results:**

We screened 1,043 MSM in 2018 and 2019, with 53.0% currently using PrEP. At screening, 370 participants (35.5%) had an STI. The most common pathogen was MG in 198 (19.0%) participants, followed by CT (n = 133; 12.8%), NG (n = 105; 10.1%) and TP (n = 37; 3.5%). Among the 370 participants with at least one STI, 14.6% (n = 54) reported STI-related symptoms. Infection prevalence was highest at anorectal site (13.4% MG, 6.5% NG, 10.2% CT). PrEP use was not statistically significant in adjusted models for STI (PR: 1.10; 95% CI: 0.91–1.32), NG/CT, only NG or only CT.

**Conclusions:**

Prevalence of asymptomatic STI was high, and PrEP use did not influence STI prevalence in MSM eligible for PrEP according to national guidelines.

## Introduction

Globally, the number of diagnoses of sexually transmitted infections (STI) has steadily increased in recent years [1]. The World Health Organization (WHO) estimated that in 2016, there were 376 million new infections with *Neisseria gonorrhoeae* (NG), *Chlamydia trachomatis* (CT), Treponema pallidum *(TP)* or *Trichomonasis vaginalis* (TV) [1]. While STI diagnoses have increased across the general population, men who have sex with men (MSM) are disproportionately affected [2]. In pooled analyses of MSM with sexual risk behaviours, the overall STI incidence rate was 84.4 new infections per 100 person-years (PY), including 64.4 infections per 100 PY in a North American cohort, 99.8 infections per 100 PY in an Australian cohort and 97.8 new infections per 100 PY in a Dutch cohort [3].

The expansion of pre-exposure prophylaxis (PrEP) against HIV, may be accompanied by a change in behaviours that impact acquisition of other STI. Individuals who use PrEP may adjust their sexual behaviours based on their reduced risk for HIV acquisition and this risk compensation could include decreased condom use and/or an increase of partner numbers compared with those not on PrEP [4]. Other studies have not found such risk compensation but have described increased STI diagnoses as a consequence of improved case finding of asymptomatic infections when PrEP users are regularly screened [5]. In a meta-analysis of 88 studies examining gonorrhoea, chlamydia and syphilis among individuals using PrEP, the prevalence of these STI was 23.9% at PrEP initiation with an incidence of 72.2 new infections per 100 PY during PrEP use, i.e. within the range of new infections among all MSM [6]. In contrast, in a cross-sectional study of MSM in Germany, PrEP use was associated with increased odds of testing positive for at least one STI excluding HIV [7].

While several studies have examined the association between PrEP use and STI, many were secondary analyses of PrEP roll-out studies and consequently only examined STI among participants who initiated PrEP and did not include a comparison with non-users. In addition, most studies only tested for STI at one or two anatomical sites with a focus on gonorrhoea, chlamydia and syphilis, while ignoring other pathogens that are common among MSM such as the emerging sexually transmitted bacterium *Mycoplasma genitalium* (MG). Our objective was to comprehensively quantify the prevalence of STI at the screening visit for entry into a cohort of MSM at risk for HIV infection and evaluate potential associations between PrEP use and STI.

## Methods

### Study procedures

The *Longitudinal Incidence Study in Subtype B-prevalent Region Among MSM at Risk for HIV Infection to Determine Feasibility of HIV Vaccine Efficacy Trials* (BRAHMS) was a prospective study conducted at 10 sites in seven major German cities (Berlin, Bochum, Cologne, Essen, Frankfurt, Hamburg and Munich) that enrolled MSM at risk of HIV infection from 4 June 2018 to 3 July 2019. Individuals were eligible to enrol if they had a non-reactive HIV test, identified as male (either at birth, chosen or intersexual), were 18–55 years-old and met either of the following two risk criteria [1]: self-reported condomless anal intercourse with at least two unique male partners known to be living with HIV or with unknown HIV status in the past 24 weeks or [2] documented history of syphilis, acute hepatitis C or rectal infection with MG, NG or CT in the past 24 weeks. Individuals were excluded from study participation if they previously participated in a candidate HIV vaccine study or were concurrently participating in any study of investigational agents for HIV prevention or treatment. Participants were allowed to enrol if they were taking approved agents for pre- or post-exposure prophylaxis, use of which was systematically documented.

At the screening visit to determine study eligibility, basic demographical data were collected and participants without HIV were counselled about PrEP. Blood was collected to test for HIV infection, active syphilis and hepatitis A, B and C. Urine and anal and pharyngeal swabs were collected to test for MG, NG, CT and TV. STI testing was performed according to standard diagnostic methods (see the Supplement for additional information on diagnostic testing). All study visits also included risk reduction counselling, provision of condoms and condom-compatible lubricants, and completion of sexual behaviour questionnaires. For these cross-sectional analyses, only data from the screening visit were included.

### Clinical and behavioural data collection

At each visit, a comprehensive medical history was taken and an extensive medical record review was performed, which included documentation of any PrEP start and stop dates. Current PrEP use was defined by the presence of a PrEP start date and indication of ongoing use in medical records. Participants without PrEP use information recorded or a stop date before the study visit were classified as not currently on PrEP. Symptoms potentially associated with an STI that occurred within the last 30 days before study participation or were ongoing were documented overall and then by each individual symptom. For these analyses, only participants experiencing symptoms on the date of the visit were categorised as symptomatic. 

At screening, participants completed a demographical and sociobehavioural questionnaire that assessed age, place of birth, education, gender identity, sexual orientation, number of male partners, condom use, recreational drug use and engagement in transactional sex. For both steady and casual partners, participants indicated the percentage of sex acts during which they were the receptive or insertive partner in the last 12 months. Sexual positioning was further classified, categorising participants as exclusively receptive, exclusively insertive or both. Condom use was assessed by asking the number of male steady or casual partners with whom the participant had condomless anal intercourse in the last 12 months. Participants who reported using a condom for anal intercourse with all male partners were classified as having consistent condom use while those reporting any condomless anal intercourse were categorised as less than consistent. Recreational drug use was assessed by asking about the last time a participant used poppers, sedatives or tranquilisers or if they had ever taken any other recreational drug. Transactional sex was defined as either having paid or been paid to have sex with a man in the last 12 months.

### Statistical analysis

Data were entered directly into the ArcGIS Survey123 platform (Esri, Redlands, United States (US)). Analyses were performed in Stata 16.0 (StataCorp, College Station, US).

Descriptive statistics used Kruskal-Wallis and Pearson chi-squared tests to compare demographical and behavioural characteristics between participants using and not using PrEP at the time of the screening visit. Log-binomial regression was used to estimate prevalence ratios (PR) and 95% confidence intervals (CI) for the association of current PrEP use and STI at the screening visit. We ran separate models examining the association of PrEP use with (i) a positive test for MG, NG, CT and/or TP, (ii) a positive NG or CT test, (iii) a positive NG test and (iv) a positive CT test. There were no cases of TV and thus TV was not included in modelling. Model selection was performed using backwards selection with a p value < 0.05. Any variable qualifying for inclusion in one model was included in all models.

### Ethical statement

The study was approved by institutional review boards of the Walter Reed Army Institute of Research, the University Duisburg–Essen and all collaborating institutions (registration number: 17–7598-BO; ClinicalTrials.gov ID: NCT03884816). All participants provided written informed consent for the study and publication of results before enrolment. The planning conduct and reporting of this study was in line with the Declaration of Helsinki, as revised in 2013.

## Results

A total of 1,043 individuals were screened for the study, with 53.0% (n = 553) using PrEP at the time of the screening visit. Among all participants, the median age was 33 years (interquartile range: 28–39), while participants currently using PrEP were slightly older than those who reported not to be on PrEP (34 vs 31 years; p < 0.001; (Table 1). The majority of participants identified as cisgender men (98.5%; n = 1,027) and as gay or homosexual (91.9%; n = 959).

**Table 1:** Select participant characteristics, by HIV pre-exposure prophylaxis usage, upon screening for entry into the BRAHMS cohort, Germany, June 2018 to July 2019 (n = 1,043)

	All participants n = 1,043		Not using PrEP n = 490		Using PrEP n = 553		p value
	n	%	n	%	n	%	
**Age (years)**							
18–29	335	32.1	202	41.2	133	24.1	**< 0.001**
30–39	482	46.2	210	42.9	272	49.2	
40–49	191	18.3	64	13.1	127	23.0	
50–55	35	3.4	14	2.9	21	3.8	
**Sex at birth**							
Male	1,042	99.9	489	99.8	553	100.0	0.29
Female	1	0.1	1	0.2	0	0.0	
**Gender identity**							
Man	1,027	98.5	482	98.4	545	98.6	0.49
Transman	1	0.1	1	0.2	0	0.0	
Other	8	0.8	3	0.6	5	0.9	
Missing	7	0.7	4	0.8	3	0.5	
**Sexual orientation**							
Gay or homosexual	959	91.9	446	91.0	513	92.8	0.21
Other^a^	74	7.1	40	8.2	34	6.1	
Missing	10	1.0	4	0.8	6	1.1	
**Place of birth**							
Other country	327	31.4	145	29.6	182	32.9	0.25
Germany	716	68.6	345	70.4	371	67.1	
**City**							
Berlin	396	38.0	186	38.0	210	38.0	
Bochum	147	14.1	65	13.3	82	14.8	**0.049**
Cologne	165	15.8	74	15.1	91	16.5	
Essen	77	7.4	46	9.4	31	5.6	
Frankfurt	102	9.8	40	8.2	62	11.2	
Hamburg	31	3.0	11	2.2	20	3.6	
Munich	125	12.0	68	13.9	57	10.3	
**Education**							
Less than secondary school	31	3.0	14	2.9	17	3.1	**0.003**
Secondary school	465	44.6	245	50.0	220	39.8	
Undergraduate degree	176	16.9	84	17.1	92	16.6	
Master’s or doctorate degree	371	35.6	147	30.0	224	40.5	
**Sexual positioning**							
Not applicable or missing	189	18.1	84	17.1	105	19.0	0.85
Exclusively receptive	36	3.5	17	3.5	19	3.4	
Exclusively insertive	33	3.2	17	3.5	16	2.9	
Both insertive and receptive	785	75.3	372	75.9	413	74.7	

PrEP: pre-exposure prophylaxis.

^a^ Other defined as bisexual, straight or heterosexual, any other term or do not usually use a term.

Statistically significant p values (p < 0.05) are shown in bold.

Among the 907 participants reporting number of male partners at the screening visit, 692 (76.3%) reported more than 10 male partners in the preceding 12 months. The number of partners differed significantly between the two groups with 81.0% of participants on PrEP reporting more than five partners (n = 448) and 75.9% of participants not on PrEP reporting more than five partners (n = 372; p < 0.001). Compared with participants not reporting current PrEP use, a higher percentage of participants reporting current PrEP use reported less than consistent condom use with a steady partner (49.2% vs 44.5%; p = 0.05) or casual partner (76.7% vs 72.4%; p < 0.001) and using poppers within the last seven days (25.5% vs 22.0%; p = 0.03). Taken together, we found a slightly higher frequency of sexual partners as well as lower condom use among PrEP users compared with non-PrEP users.

### High frequency of sexually transmitted infections among men who have sex with men

**Figure 1:**
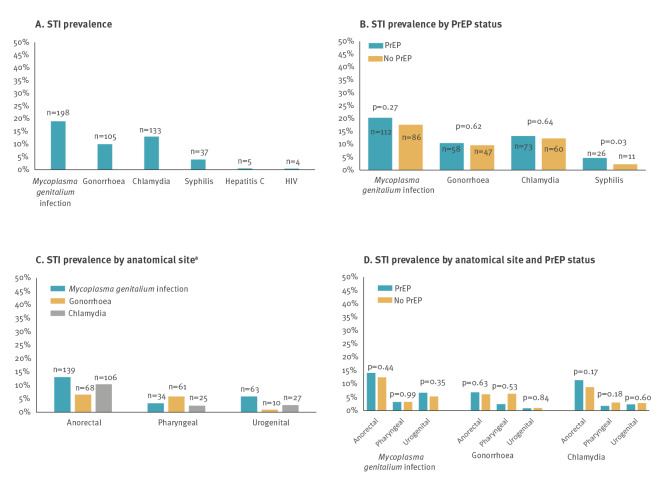
Prevalence of sexually transmitted infections, in relation to anatomical site and use of HIV pre-exposure prophylaxis, Germany, June 2018–July 2019 (n = 1,043)

PrEP: pre-exposure prophylaxis; STI: sexually transmitted infection.

^a ^Several participants tested positive for STI at multiple anatomical sites.

At the screening visits, we detected a high frequency of STI among all participants. Overall, 35.5% of participants had an STI (n = 370), including 27.3% (n = 285) who had a single STI, 7.6% (n = 79) with two STI, 1.1% (n = 11) with three STI and one individual with four STI. The most commonly observed pathogen was MG in 19.0% (n = 198) of participants, followed by CT (12.8%; n = 133), NG (10.1%; n = 105) and TP (3.5%; n = 37) (Figure 1A). Prevalence of HIV (< 1%, n = 4), hepatitis B (< 1%; n = 2) and hepatitis C (< 1%; n = 5) was low and there were no participants with acute hepatitis A or with a TV infection. The most common simultaneously occurring infections were with NG and CT (2.2%; n = 23), followed by NG and MG (2.1%; n = 22) and CT and MG (1.7%; n = 18). There were an additional eight participants with MG and TP, five participants with CT and TP and two participants with NG and TP. Ten participants had MG, NG and CT, and one participant had MG, CT and TP. 

### Prevalence of sexually transmitted infections by anatomical site

**Figure 2:**
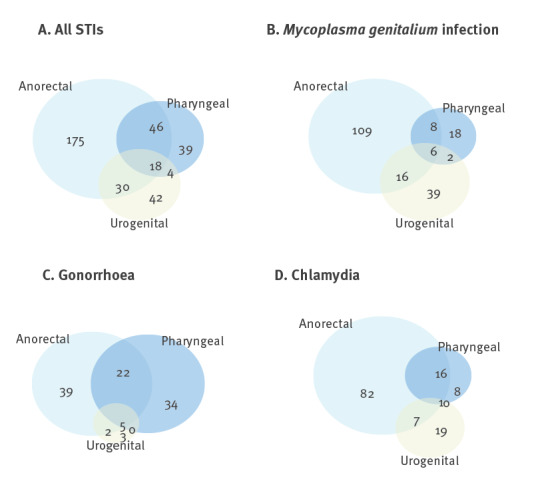
Number of prevalent diagnoses of sexually transmitted infections by anatomical site, Germany, June 2018–July 2019 (n = 1,043)

The prevalence of STI was highest at the anorectal site (25.9%) followed by pharyngeal (10.3%) and urethral (9.0%) infections. The most common anorectal infection was MG, occurring in 13.4% of the participants, followed by NG (6.5%) and CT (10.2%; Figure 1). Examining the overlap of STI by anatomical site, 1.7% of participants had an STI at all three sites, while 6.1% tested positive for STI at both anorectal and pharyngeal sites (Figure 2). These data indicate that the same or different STI can occur simultaneously at different anatomical sites.

'All STI' was defined as at least one of the following: *Mycoplasma genitalium* infection, gonorrhoea or chlamydia. Participants were classified as having a diagnosis at an anatomical site if they tested positive for any pathogens looked at in this study. For example a participant who had pharyngeal gonorrhoea and anorectal chlamydia would be classified as pharyngeal and anorectal in the 'All STI' Venn diagram. Participants with missing data for one of the anatomical sites were assumed negative at the site.

### Low level of recorded symptoms among individuals with sexually transmitted infections 

We assessed the symptoms of the 370 participants with at least one STI. Surprisingly, only 14.6% (n = 54) had symptoms. An additional 71 participants reported symptoms but did not test positive for any STI. The most frequently reported symptoms were sore throat (n = 15), pain or burning during urination (n = 9), and penile, urethral or neovaginal discharge (n = 8). Five participants reported painful swallowing, itching or other discomfort in the perianal area, pain or burning during defecation or difficulty urinating. Among the 54 participants reporting symptoms, 34 reported one symptom while 10 reported two, eight reported three, one participant reported four and one participant reported six symptoms. 

### Use of pre-exposure prophylaxis and sexually transmitted infections 

We asked the question whether PrEP use was associated with increased levels of STI. Descriptively, only syphilis positivity differed significantly by PrEP use (4.7% vs 2.3%; p = 0.03) (Figure 1B) and there were no significant differences in STI by anatomical site and PrEP use (Figure 1D). None of the four participants with HIV reported PrEP use, while two participants with hepatitis B and two with hepatitis C were on PrEP. In the unadjusted model, PrEP use was not significantly associated with overall prevalence of any STI (MG, NG, CT or TP; PR: 1.17; 95% CI: 0.99–1.39; Supplementary Table S1 provides unadjusted PR) and was not statistically significant in the adjusted model (adjusted PR: 1.10; 95% CI: 0.91–1.32) (Table 2). There was no significant association between PrEP use and NG/CT in the unadjusted and adjusted models (Table 2, Supplementary Table S2 provides additional unadjusted PR for the association between select factors and NG/CT) nor in the model examining only NG (Table 2, Supplementary Table S3 provides unadjusted PR for NG) or only CT (Table 2). Taken together, we did not observe significant differences in STI in a point prevalence analysis between PrEP users and non-PrEP users.

**Table 2:** Adjusted prevalence ratios for HIV pre-exposure prophylaxis usage and other factors potentially associated with *Mycoplasma genitalium* infection, gonorrhoea, chlamydia or syphilis, Germany, June 2018–July 2019 (n = 1,043)

	Any STI^a^		Gonorrhoea or chlamydia		Gonorrhoea		Chlamydia	
	aPR	95% CI	aPR	95% CI	aPR	95% CI	aPR	95% CI
**PrEP**								
Not using PrEP	Reference							
Using PrEP	1.10	0.91–1.32	1.00	0.76–1.32	0.92	0.61–1.37	1.06	0.74–1.51
**Education**								
Less than a university degree	Reference							
University degree or higher	0.88	0.73–1.05	0.82	0.62–1.07	0.83	0.56–1.24	0.78	0.55–1.13
**Number of partners (last 12 months)**								
≤ 5	Reference							
> 5	2.01	1.24–3.25	1.74	0.93–3.27	2.11	0.79–5.60	1.60	0.73–3.52
**Sexual positioning**								
Not applicable or missing information	0.95	0.62–1.44	0.54	0.24–1.19	0.90	0.03–2.71	0.25	0.05–1.17
Exclusively receptive	Reference							
Exclusively insertive	0.64	0.36–0.92	0.20	0.05–0.83	0.43	0.09─2.09	0.19	0.02–1.54
Both insertive and receptive	0.80	0.62–1.05	0.61	0.37–1.01	0.68	0.29–1.58	0.77	0.36–1.64

aPR: adjusted prevalence ratio; CI: confidence interval; PrEP: pre-exposure prophylaxis.

^a^ Any sexually transmitted infection defined as at least one of the following: *Mycoplasma genitalium* infection, gonorrhoea, chlamydia or syphilis.

## Discussion

In this cohort of predominantly MSM at risk for HIV in Germany, STI prevalence was high at baseline screening, especially with the emerging sexually transmitted bacterial infection MG. Compared with a similar cohort of MSM in France enrolled from 2015 to 2016, MG prevalence in our cohort was nearly twice as high [8]. A meta-analysis of MG among MSM globally reported a prevalence of 3.2% (95% CI: 2.1–5.1%) [9]. However, other studies have found similar prevalence as ours with 15% prevalence among asymptomatic MSM in Australia [10] and 17% among care-seeking men in the US [11]. Our findings were in line with another cross-sectional analysis from 2018 of MSM on PrEP in Germany with a 20% prevalence of MG, although they had a slightly lower prevalence among MSM not on PrEP [7]. MG is increasingly recognised as a cause of non-gonococcal, non-chlamydial urethritis in men [12,13] as well as asymptomatic rectal infection and symptomatic proctitis among MSM [14,15]. There is in vitro evidence that MG may facilitate HIV infection through mucosal disruption [16,17], an inflammatory response that recruits HIV-susceptible cells to the mucosal surface [18], and direct enhancement of HIV replication [19,20]. Routine screening for MG is not currently recommended in Germany. Given the high prevalence of asymptomatic disease, potential for morbidity and enhanced risk of HIV transmission, routine screening for MG could be considered for German MSM, although management of asymptomatic MG infection is unclear and requires further investigation. In this context, the high levels of antimicrobial resistance of MG in MSM against macrolides and increasing resistance against quinolones are of special importance [21,22].

We also found a high prevalence of gonorrhoea and chlamydia. A meta-analysis of 12 studies found a similar CT prevalence of 10.8%, although the prevalence varied slightly more by anatomical site as in our cohort (4% urogenital prevalence and 8.5% rectal) [6]. Another German study found a similar prevalence of NG (8.9%) but a lower prevalence of CT (9.9%), with a similar distribution across anatomical sites as seen in our study [7]. In a care-seeking sample of MSM in Portugal, prevalence of NG was 10.7% and prevalence of CT was at 7.6% less than half of the prevalence in the BRAHMS cohort [23]. The discrepancy in CT prevalence may be due in part to the different inclusion criteria for the respective studies as the Portuguese study included any care-seeking individual who had results for CT/NG tests at all three anatomical sites. Lower prevalence of STI in other studies may also be due to testing only one or two anatomical sites. Systematic testing for STI at all three anatomical sites among participants in our study provides a comprehensive understanding of the burden of infection and the cases that may be missed when only one or two anatomical sites are evaluated. Testing only at the anorectal site would have missed 23% of infections in this study.

We found a comparably low prevalence of syphilis, hepatitis B and C and no cases of hepatitis A or of TV infection. In a meta-analysis of individuals using PrEP, pooled prevalence of syphilis (5.0%) was similar to our findings but the prevalence of hepatitis A (5.4%), hepatitis B (1.3%), hepatitis C (2.0%) and TV (5.9%) was higher [6]. Co-infection with multiple STI was less common in our study than in similar cohorts elsewhere: In a study of MSM in Australia, 13% of participants with CT also had MG and 14% were coinfected with MG and NG [24]. Among care-seeking MSM in London, 7% had CT/NG coinfections [25]. The difference in prevalence in these various groups may be due to the criteria for inclusion in the studies as some involved only care-seeking or symptomatic individuals.

We also found that PrEP use had no association with STI in cross-sectional analyses. Other cohort studies have found an increase in STI after initiation of PrEP [3,26,27]. The mechanism for these findings could relate to increased screening among individuals on PrEP or HIV risk compensation behaviours beyond condom use such as increasing partner numbers. In another study of German MSM, PrEP use was associated with twice the odds of any STI (95% CI: 1.5–2.7) [7]. While both studies examined care-seeking MSM attending MSM-friendly practices, BRAHMS recruited individuals reporting sexual behaviours with increased risk for acquiring STI or with recent known history of STI, so we expected the differences in sexual risk behaviour between PrEP and non-PrEP users in our study to be less pronounced. A randomised controlled trial in 13 sexual health clinics in England assessed the effect of risk compensation in users of PrEP and found no difference in STI occurrence [28]. We also found no significant association between PrEP use and NG/CT. This finding is in agreement with a clinic-based study among MSM in the US, where CT prevalence remained constant at 9.6% before and after PrEP initiation and NG prevalence remained stable (11.7% vs 10.3%) before and after PrEP initiation [5]. We found small differences in engaging in sexual risk behaviours comparing PrEP users to non-users; this may be due to the inclusion criteria and the risk profile that was required to fulfil eligibility for this study. In addition, participants in our study were engaged in care at centres specialising in providing care to sexual and gender minorities at risk for HIV; good counselling practices may have impacted the lack of risk compensation observed in our study as compared with others. Our study makes it clear that regular screening for STI is an important component of caring for behaviourally vulnerable to STI German men regardless of PrEP use. Concern about risk compensation should not be a barrier to PrEP use in men with behavioural risk factors for HIV acquisition.

The high prevalence of STI in a mostly asymptomatic cohort of MSM suggests the importance of regular STI screening. Testing only symptomatic individuals would have missed 85% of infections in this population. Other studies have similarly found a predominance of asymptomatic infections among MSM [7,29,30]. Effective screening practices should also include anatomically appropriate screening and testing at multiple anatomical sites. While the prevalence was highest among anorectal STI, we also found pathogens present in both pharyngeal and urogenital samples, highlighting the need for screening at all sites. In a meta-analysis of 12 STI and PrEP use studies, among participants with positive NG tests, 13.8% had both positive anorectal and pharyngeal tests while 2.5% had positive pharyngeal, anal and urogenital tests [23]. The decision to treat asymptomatic infections with MG, NG and CT needs to weigh advantages in terms of STI sequelae with the potential damages to the microbiome caused by frequent use of antibiotics.

Strengths of this study include the extensive medical record review of PrEP data, medical history and testing for a wide array of STI at multiple anatomical sites. However, this study is not without limitations. Firstly, the cross-sectional nature of our analyses does not allow for any comment on causality in the association between PrEP use and STI. However, these analyses provide a snapshot of the STI prevalence among MSM at risk for HIV who were engaged in routine care before entry into a cohort study that included enhanced STI testing. Secondly, results for lymphogranuloma venereum were not available at the time of this analysis which may explain some of the reported rectal symptoms. Thirdly, we only present symptom information at the time of STI testing which may underestimate actual symptomatic infections if symptoms in the previous weeks were not serious enough to seek medical advice. Fourthly, the risk profile required to enrol in BRAHMS, allowing only for men at high risk of HIV infection, and the active counselling as part of study procedures may make these findings not generalisable to other populations, but can serve as a good basis for diagnostic and treatment decisions for MSM eligible for PrEP. Finally, the clinics from which participants were enrolled cater to gender and sexual minorities and have expertise in HIV prevention and treatment for these populations.

## Conclusion

We found a high prevalence of asymptomatic rectal STI in a cohort of behaviourally vulnerable MSM as well as a high prevalence of STI in both urogenital and oropharyngeal sites, highlighting a need for regular and comprehensive screening at several sites.

## Supplementary Data

142000 Supplement
