# Supplementary material for: HIV pre-exposure prophylaxis was associated with no impact on sexually transmitted infection prevalence in a high-prevalence population of predominantly men who have sex with men, Germany, 2018 to 2019
Source: Euro Surveill. 2022 Apr 7;27(14):2100591. doi: 10.2807/1560-7917.ES.2022.27.14.2100591 (PMC8991735; doi:10.2807/1560-7917.ES.2022.27.14.2100591)
Supplement: Supplement [file 21-00591_ESBER_Supplement.pdf]

This supplementary material is hosted by *Eurosurveillance* as supporting information alongside the article “HIV pre-exposure prophylaxis was associated with no impact on sexually transmitted infection prevalence in a high-prevalence population of predominantly men who have sex with men in Germany, 2018-2019”, on behalf of the authors, who remain responsible for the accuracy and appropriateness of the content. The same standards for ethics, copyright, attributions and permissions as for the article apply. Supplements are not edited by *Eurosurveillance* and the journal is not responsible for the maintenance of any links or email addresses provided therein.

Diagnosis of HIV and other STIs

All participants were tested for HIV at the point-of-care using the fourth-generation Alere HIV Combo rapid test (Abbott GmbH & Co. KG, Wiesbaden, Germany). Centralized confirmatory HIV testing was performed using the fourth-generation Alinity I HIV Ag/Ab Combo assay (Abbott GmbH & Co. KG, Wiesbaden, Germany) and the Aptima HIV-1 Quant Dx assay (Hologic, Marlborough, MA, USA).

Hepatitis A status was determined using Alinity i HAVAb IgG and IgM Reagent kits. Hepatitis B infection was diagnosed using the Alinity HBsAg Qualitative II assay (Abbott Ireland, Sligo, Ireland) to detect surface antigen. Hepatitis C was diagnosed using the Aptima HCV Quant Dx assay (Hologic, Marlborough, MA, USA) to detect and quantify HCV RNA. Testing for active syphilis was performed using CMIA-test and confirmation testing was conducted using TPPA, FTABS-G/M, RPR, and VDRL. All testing was performed according to manufacturer instructions.

Voided urine, anal swabs, and oropharyngeal swabs were collected from all participants. These specimens were tested for CT and NG using the Aptima Combo 2, MG using the Aptima Mycoplasma genitalium Assay, and TV using the Aptima Trichomonas vaginalis Assay (all assays by Hologic, Wiesbaden, Germany & Bedorf, US).

Supplemental Table 1: Unadjusted and Adjusted Prevalence Ratios for PrEP use and other factors potentially associated with any STI (syphilis, gonorrhea, chlamydia or Mycoplasma genitalium)

	Prevalence Ratio	95% Confidence Interval	Adjusted Prevalence Ratio	95% Confidence Interval
**PrEP**				
Not Using PrEP	Ref		-	
Using PrEP	1.17	0.99-1.39	1.10	0.91-1.32
**Age (years)**				
18-29	Ref			
30-39	0.85	0.71-1.02		
40-49	0.86	0.67-1.09		
50-55	0.65	0.36-1.15		
**Sexual orientation**				
Gay or homosexual	Ref			
Other	1.07	0.79-1.45		
**Place of birth**				
Other Country	Ref			
Germany	0.91	0.76-1.07		
**City**				
Essen	Ref			
Frankfurt	1.03	0.67-1.57		
Hamburg	1.19	0.69-2.06		
Munich	0.81	0.53-1.26		
Cologne	1.29	0.89-1.86		
Berlin	1.21	0.86-1.71		
Bochum	0.86	0.57-1.30		
**Education**				
Less than a college degree	Ref		-	
Earned a bachelor degree or higher	0.87	0.74-1.02	0.88	0.73-1.05
**Number of partners**				
≤5	Ref		-	
>5	1.98	1.22-3.21	2.01	1.24-3.25
**Condomless anal intercourse (all partners)**				
Less than consistent condom use	Ref			
Consistent condom use	0.54	0.32-0.90		
Not applicable or missing	1.18	0.97-1.43		
**Sexual positioning**				
Not applicable or missing	0.87	0.59-1.28	0.95	0.62-1.44
Exclusively Receptive	Ref		-	
Exclusively Insertive	0.38	0.17-0.86	0.64	0.36-0.92
Both Insertive and Receptive	0.72	0.51-1.04	0.80	0.62-1.05

Supplemental Table 2: Unadjusted and Adjusted Prevalence Ratios for Association between PrEP use and Gonorrhea or Chlamydia at Screening Visit

	Prevalence Ratio	95% Confidence Interval	Adjusted Prevalence Ratio	95% Confidence Interval
**PrEP**				
Not Using PrEP	Ref		-	
Using PrEP	1.07	0.84-1.37	1.00	0.76-1.32
**Age (years)**				
18-29	Ref			
30-39	0.93	0.71-1.23		
40-49	0.93	0.65-1.32		
50-55	0.68	0.29-1.58		
**Sexual orientation**				
Gay or homosexual	Ref			
Other	1.10	0.70-1.73		
**Place of Birth**				
Other Country	Ref			
Germany	0.91	0.70-1.18		
**City**				
Essen	Ref			
Frankfurt	0.93	0.47-1.81		
Hamburg	1.34	0.59-3.03		
Munich	0.90	0.47-1.72		
Cologne	1.74	1.01-3.02		
Berlin	1.20	0.70-2.04		
Bochum	0.93	0.50-1.74		
**Education**				
Less than a college degree	Ref		-	
Earned a bachelor degree or higher	0.82	0.64-1.05	0.82	0.62-1.07
**Number of partners**				
≤5	Ref		-	
>5	1.67	0.89-3.14	1.74	0.93-3.27
**Condomless anal intercourse (all partners)**				
Less than consistent condom use	Ref			
Consistent condom use	0.31	0.12-0.82		
Not applicable or missing	1.13	0.84-1.52		
**Sexual positioning**				
Not applicable or missing	0.73	0.42-1.27	0.54	0.24-1.19
Exclusively Receptive	Ref		-	
Exclusively Insertive	0.30	0.09-0.95	0.20	0.05-0.83
Both Insertive and Receptive	0.60	0.36-1.01	0.61	0.37-1.01

Supplemental Table 3: Unadjusted and Adjusted Prevalence Ratios for Association between PrEP use and Gonorrhea at Screening Visit

Prevalence Ratio	95% Confidence Interval	Adjusted Prevalence Ratio	95% Confidence Interval	Prevalence Ratio
**PrEP**				
Not Using PrEP	Ref		-	
Using PrEP	1.09	0.76-1.57	0.92	0.61-1.37
**Age (years)**				
18-29	Ref			
30-39	0.86	0.57-1.30		
40-49	0.81	0.47-1.39		
50-55	1.29	0.54-3.07		
**Sexual orientation**				
Gay or homosexual	Ref			
Other	1.22	0.64-2.32		
**Place of Birth**				
Other Country	Ref			
Germany	0.81	0.55-1.18		
**City**				
Essen	Ref			
Frankfurt	1.32	0.40-4.35		
Hamburg	3.10	0.89-10.81		
Munich	1.39	0.44-4.35		
Cologne	3.54	1.29-9.71		
Berlin	2.04	0.75-5.53		
Bochum	1.05	0.33-3.39		
**Education**				
Less than a college degree	Ref		-	
Earned a bachelor degree or higher	0.89	0.62-1.28	0.83	0.56-1.24
**Number of partners**				
≤5	Ref		-	
>5	1.98	0.74-5.24	2.11	0.79-5.60
**Condomless anal intercourse (all partners)**				
Less than consistent condom use	Ref			
Consistent condom use	0.83	0.54-1.29		
Not applicable or missing	0.26	0.06-1.08		
**Sexual positioning**				
Not applicable or missing	0.86	0.35-2.10	0.90	0.30-2.71
Exclusively Receptive	Ref		-	
Exclusively Insertive	0.42	0.09-2.04	0.43	0.09-2.09
Both Insertive and Receptive	0.67	0.29-1.55	0.68	0.29-1.58
